# Combination of curcuminoid and collagen marine peptides for healing diabetic wounds infected by methicillin-resistant *Staphylococcus aureus*

**DOI:** 10.14202/vetworld.2024.933-939

**Published:** 2024-04-29

**Authors:** Dwi Ardyan Syah Mustofa, Farhan Dio Sahari, Syifa Aulia Pramudani, Alifia Brilliani Hidayah, Shabrina Farras Tsany, Siti Isrina Oktavia Salasia

**Affiliations:** 1 Department of Clinical Pathology, Faculty of Veterinary Medicine, Universitas Gadjah Mada, Yogyakarta, Indonesia; 2 Department of Pharmaceutics, Faculty of Pharmacy, Universitas Gadjah Mada, Yogyakarta, Indonesia; 3 Department of Physiology, Faculty of Medicine, Public Health and Nursing, Universitas Gadjah Mada, Yogyakarta, Indonesia

**Keywords:** collagen marine peptides, curcuminoids, diabetic, methicillin-resistant *Staphylococcus aureus*, wound

## Abstract

**Background and Aim:**

The high prevalence of diabetes mellitus in Indonesia indirectly reflects the high risk of developing chronic wounds that are susceptible to infection. Methicillin-resistant *Staphylococcus aureus* (MRSA) is an infectious pathogen that is resistant to various antibiotics. Therefore, antibiotic therapy is ineffective enough to treat chronic hyperglycemic wounds caused by MRSA infection. Curcuminoids have anti-inflammatory and antibacterial effects by inhibiting the enzymatic pathways involved in the pathogenesis of inflammation. Collagen is a tissue regeneration inducer. The combination of these two ingredients is expected to be an alternative therapy for MRSA-infected hyperglycemic chronic wounds without the risk of antibiotic resistance. The aim of this study was to investigate the efficacy of hydrolacin-gel in wound healing and inhibiting the growth of MRSA bacteria, as well as to determine the optimal concentration of curcuminoids combined with collagen marine peptides (CMPs).

**Materials and Methods:**

Hydrolacin-gels were prepared by homogenizing curcuminoid nanoemulsions and CMPs. The evaluation of preparation includes stability tests and antibacterial activity tests. Wound diabetic mice were treated with various combinations of curcuminoid and CMPs. Wound healing was observed based on malondialdehyde levels as a marker of oxidative stress and histopathological changes in the skin wound.

**Results:**

Hydrolacin-gel was formulated by combining curcuminoid nanoemulsion (more water soluble) and CMPs, with the ratio of formula 1 (1:2, curcuminoid 43.3 mg and CMPs 5.58 mg), formula 2 (1:1, curcuminoid 86.8 mg and CMPs 3.72), and formula 3 (2:1, curcuminoid 130.2 mg and CMPs 1.87 mg) calculated based on the effective dose of curcuminoid 200 mg/kg body weight (BW) and CAMPs 0.9 g/kg BW. Hydrolacin-gel had a potential antibacterial activity against MRSA. Hydrolacin-gel induced wound tissue repair and reduced oxidative stress caused by inflammation in diabetic-infected MRSA. Hydrolacin-gel could be used for healing MRSA-infected diabetic wounds, especially formula 3 with the ratio of curcuminoid: CMPs = 2:1.

**Conclusion:**

Hydrolacin-gel combining curcuminoid nanoemulsion and CMPs effectively inhibited the inflammatory process and increased re-epithelialization in MRSA-infected diabetic wound healing. Hydrolacin-gel with curcuminoid (130.2 mg) and CMPs (1.87 mg) at a concentration ratio of 2:1 appeared to be the best formula against MRSA infection in diabetic wounds.

## Introduction

Hyperglycemia is characterized by elevated blood sugar levels that lead to harmful metabolic effects [1]. Indonesia ranks second among countries with the highest prevalence of hyperglycemia with 19.5 million cases, which is expected to increase to 28.6 million by 2045 [2]. With the epidemiological status of hyperglycemia, the risk of chronic wound occurrence in Indonesia has also risen due to hyperglycemia being a contributing factor [3]. Chronic wounds caused by hyperglycemia are susceptible to infections that may ultimately lead to amputation and mortality. Methicillin-resistant *Staphylococcus aureus* (MRSA) is a bacterial strain that dominates infections in chronic wounds [4].

In general, chronic hyperglycemic wounds infected with MRSA require surgery and antibiotic administration. However, surgery tends to be a last resort and is cost-intensive, whereas antibiotic administration is ineffective due to MRSA resistance [5]. Therefore, innovative alternative therapies, such as curcuminoids and collagen, need to be developed to address chronic hyperglycemic wounds and simultaneously overcome MRSA antibiotic resistance with a more economical and effective approach. Curcuminoids have the potential to treat MRSA-infected wounds because of their anti-inflammatory and antibacterial properties. Curcuminoids inhibit cyclooxygenase-2 (COX-2) and lipoxygenase (LOX), which are crucial enzymes in the inflammatory process, while accelerating re-epithelialization, cell proliferation, and collagen synthesis in wound healing [6]. Collagen serves as a biomaterial that facilitates wound healing. The application of collagen to wounds can enhance angiogenesis and tissue repair, reduce edema, and boost metabolic activity in granulation tissue [7].

The bioavailability of curcuminoid and collagen can be optimized by processing them into nanoemulsions, which are subsequently transformed into a hydrolacin-gel formulation. Hydrolacin-gel aims to maximize the efficacy of curcuminoid and collagen compounds, presenting a potential alternative therapy for chronic hyperglycemic wound healing and addressing the issue of MRSA antibiotic resistance.

## Materials and Methods

### Ethical approval

The Animal Care and Use Committee, Faculty of Veterinary Medicine, Universitas Gadjah Mada, approved all procedures performed in this study (No. 79/EC-FKH/Eks./2023).

### Study period and location

This study was conducted from June to October 2023 in the Clinical Pathology Laboratory, Faculty of Veterinary Medicine, Laboratory of Pharmacognosy and Laboratory of Pharmaceutica, Faculty of Pharmacy, Universitas Gadjah Mada, Indonesia.

### Preparation of curcuminoid nanoemulsion

Turmeric rhizomes (*Curcuma longa*) powder was obtained from Herbal Medicine House (Yogyakarta, Indonesia). The powder was sieved through a 40-mesh sieve and macerated for 3 days in 96% ethanol. To obtain the viscous curcuminoid extract [8], the macerate was filtered and concentrated with a rotary vacuum evaporator (IKA Works [Asia], Malaysia) at a temperature of 40°C. The extract was then homogenized with Tween 80, virgin coconut oil, and polyethylene glycol (PEG 400) surfactants at 250× *g* for 10 min [9]. The average nanoemulsion droplet size was analyzed using a Zetasizer Nano SZ (Malvern Panalytical Ltd., Malvern, UK), as described by Jiang and Charcosset [10].

### Preparation and formulation of hydrolacin-gel

Hydrolacin was prepared by combining curcuminoid nanoemulsion and collagen marine peptides (CMPs, California Gold, USA). Curcuminoid in the form of nanoemulsions is more water soluble when combined with CMPs. The gel was prepared using gelatin, sodium carboxymethyl cellulose (CMC-Na), and Carbopol (Sigma, Aldrich, Germany), and homogenized with triethanolamine to form the gel base [11]. Methyl paraben and propyl paraben were dissolved in propylene glycol (Sigma) and then added to the gel base, followed by the addition of curcuminoid nanoemulsion and CMPs with concentration variations of formula 1 (1:2, curcuminoid 43.3 mg and CMPs 5.58 mg), formula 2 (1:1, curcuminoid 86.8 mg and CMPs 3.72 mg), and formula 3 (2:1, curcuminoid 130.2 mg and CMPs 1.87. The ingredients were homogenized to prepare the hydrolacin-gel. The curcuminoid and CMP concentrations used in the hydrolacin-gel formulation were based on effective doses of 200 mg/kg body weight (BW) (curcuminoid) and 0.9 g/kg BW (CMPs) for inflammation treatment [6, 7]. Table-1 summarizes the calculation of this formula [6, 7].

**Table-1:** Formulation of hydrolacin-gel.

Ingredients	Formula 1 (mg)	Formula 2 (mg)	Formula 3 (mg)
Carbopol	1.67	3.34	5.00
Gelatin	0.83	1.67	2.50
CMC-Na	2.40	4.80	7.20
Propylene glycol	4	4	4
Triethanolamine	6	6	6
Glycerol	25	25	25
Methyl paraben	0.30	0.30	0.30
Prophyl paraben	0.06	0.06	0.06
Curcuminoid nanoemulsion*	43.30	86.80	130.20
Collagen marine peptides**	5.58	3.71	1.86
Aquadest	ad 100 mL	ad 100 mL	ad 100 mL

* Based on the effective dose of curcuminoid (200 mg/kg BW);

** Based on the effective dose of collagen marine peptides (0.9 g/kg BW) according to previous studies Qureshi *et al*. [6], Thahir and Wahyuni [7]. BW=Body weight

### Characterization of hydrolacin-gel

According to a previous study by Rahmadani *et al*. [12], the effectiveness and stability of hydrolacin-gel were evaluated using various tests such as organoleptic, pH, viscosity, homogeneity, syneresis, spreadability, and adhesion properties. The physical characteristics of hydrolacin-gel formulation were obtained from physical stability testing using the freeze-thaw cycling method to assess the stability of hydrolacin-gel formulation in maintaining its physical characteristics over three cycles.

### Antibacterial activity test of hydrolacin-gel against MRSA

Antibacterial activity on Mueller Hinton agar (MHA) was tested using the well diffusion method. MRSA culture was poured onto solid MHA with a well-forming agent and introduced into wells with various formulas of hydrolacin-gel and clindamycin used as a control. Hydrolacin-gel’s antibacterial activities were indicated by the inhibition zone around the wells after incubation for 24 h at 37°C [13].

### MRSA-infected wound diabetic animal model

Twenty-five male Wistar rats (8 weeks old; BW, 150–200 g) obtained from the Nutrition Laboratory, Inter University Center, Universitas Gadjah Mada, Indonesia, were used as experimental animals. The rats were acclimatized for 1 week and fasted 1 night before treatment. Rats were induced to become diabetic by intraperitoneal injection of streptozotocin (45 mg/kg BW) and niacinamide (110 mg/kg BW). Rat blood was taken from the retroorbital plexus after intramuscular anesthesia with a combination of ketamine (50 mg/kg BW) and xylazine (5 mg/kg BW) to monitor glucose levels up to 200 mg/dL. After anesthesia, the dorsal subcutaneous area of the rats was wounded with a 5-mm biopsy punch and infected with 50 L of 0.5 McFarland MRSA suspension [14].

### Experimental design

The 25 Wistar rats were divided into five treatment groups as negative control (K) without treatment, positive control (K+) treated with commercial clindamycin phosphate 1%, P1 group treated with hydrolacin-gel formula 1 (curcuminoid: CMPs = 2:1), P2 group treated with hydrolacin-gel formula 2 (curcuminoid: CMPs = 1:1), and P3 group treated with hydrolacin-gel formula 3 (curcuminoid: CMPs = 1:2). Hydrolacin-gel was topically applied on the rat’s wound once daily for 14 days [14].

### Measurement of malondialdehyde (MDA)

At the end of the experiments (14^th^ day), rats were euthanized by intraperitoneal overdose of ketamine (300 mg/kg BW) and xylazine (30 mg/kg BW) to observe MDA and histopathological changes of wound tissue. MDA was measured using the competitive enzyme-linked immunosorbent assay (ELISA) method. The wound skin tissue (1 × 1 cm) was scraped, homogenized with 9 mL of phosphate-buffered saline, and centrifuged at 5000× *g* for 5 min at 2°C–8°C [15]. The MDA concentration was adjusted according to the Rat MDA ELISA kit procedure (Elabscience, USA). Tissue samples and ELISA kit standards were labeled with biotin-labeled antibodies, and 3,3’,5,5’ tetramethylbenzidine (TMB) substrates were added. A spectrophotometer was used to measure the optical density of the samples at a wavelength of 450 nm [16].

### Histopathological examination

Wound skin tissue, including normal skin control, was cut 0.5–1 cm from the wound edge. Skin sections were placed in a 10% neutral buffered formalin (NBF) solution for 15–24 h, stained using H&E, and observed under a light microscope (Olympus, Tokyo, Japan) [14].

### Statistical analysis

Data analysis was performed using Statistical Package for the Social Sciences Statistics 25 software (IBM Corp., NY, USA). Quantitative data analyses were performed using paired t-tests and one-way analysis of variance, followed by *post-hoc* least significant difference tests. A p < 0.05 was considered to indicate statistically significant differences. Histopathological changes in rat skin tissues were descriptively analyzed and compared with the control.

## Results

### Curcuminoid extraction and nanoemulsion

Using the maceration method, 300 g of small-sized dry turmeric powder was obtained after sieving into a 40-mesh. A total of 16.28 g of curcuminoid with 5.43% yield was collected from the ethanol extract. The average droplet size of the curcuminoid nanoemulsion was 14.68 nm with a polydispersity index (PdI) of 0.389.

### Characteristics of hydrolacin-gel

Table-2 shows the physical characteristics of the hydrolacin-gel formulation. The physical properties of hydrolacin-gel before and after stability testing are presented in Table-3. No significant shifts were observed in pH, spreadability, syneresis, and viscosity values of hydrolacin-gel before and after the stability test (p > 0.05).

**Table-2:** Characteristics of hydrolacin-gel.

Response	Formula 1	Formula 2	Formula 3
Organoleptic	Dark yellow, in the form of viscous gel, easy to apply		
Viscocity	25701.7	26385.5	21855.5
pH	7	7	7
Spreadability (cm)	5.1	5.2	5.08
Syneresis (%)	10.57	7.09	6.99

**Table-3:** Comparison of the physical properties of hydrolacin-gel before and after stability testing.

Response	Before stability tests	After stability tests	p-value
Organoleptic	Dark yellow, in the form of viscous gel, easy to apply	Dark yellow, in the form of viscous gel, easy to apply	-
Viscocity	25033.22	22834.66	0.14
pH	7	6.8	0.42
Spreadability (cm)	5.08	5.01	0.91
Syneresis (%)	8.25	9.44	0.80

### Antibacterial activity of hydrolacin-gel against MRSA

**Figure-1:**
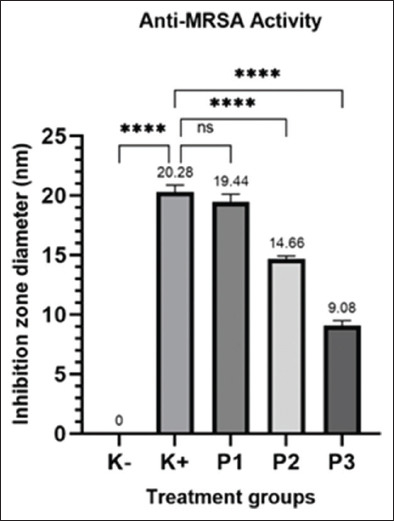
MRSA antibacterial activity test results; ****: significant difference, ns: no significant difference. MRSA=Methicillin-resistant *Staphylococcus aureus*.

The antibacterial activity of hydrolacin-gel was demonstrated by the presence of bacterial diameter inhibition zones on the MHA media. The larger the diameter of the inhibition zone, the greater the antibacterial activity. Significant differences were observed in the diameter of the inhibition zones among the treatment groups (p < 0.05), as depicted in Figure-1. The diameter of the K+ (clindamycin) inhibition zone was 20.28 mm, indicating MRSA sensitivity. Compared with the control clindamycin, P1 (formula 1 Hydrolacin-gel), P2 (formula 2 Hydrolacin-gel), and P3 (formula 3 hydrolacin-gel) had diameter zones of 19.44 mm, 14.66 mm, and 9.08 mm, respectively, indicating potential antibacterial activity against MRSA, with formula 1 of Hydrolacin-gel showing the most effective action in inhibiting MRSA growth.

### Measurement of the wound skin area of hyperglycemic rats infected with MRSA

**Figure-2:**
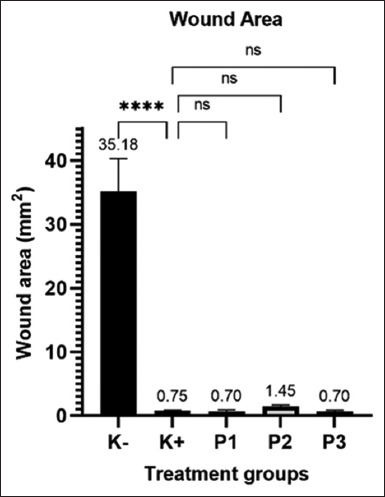
Measurement result of the area of MRSA-infected-hyperglycemic chronic-wound; ****: Significant difference, ns: no significant difference. MRSA=Methicillin-resistant *Staphylococcus aureus*.

The wound area measurements are presented in Figure-2. There was a significant difference between the K+ (clindamycin control group), P1 (formula 1 hydrolacin-gel), P2 (formula 2 hydrolacin-gel), and P3 (formula 3 hydrolacin-gel) groups with wound area sizes of 0.75 mm^2^, 0.70 mm, 1.45 mm, and 0.70 mm, respectively, compared to the K (diabetic group) with a wound area size of 35.18 mm. These results demonstrate that hydrolacin-gel with formulas 1, 2, and 3 accelerated the wound closure process by up to 97.87%, similar to that of the clindamycin group, indicating expedites the healing wound process in MRSA-infected diabetic patients.

### Measurement of MDA

**Figure-3:**
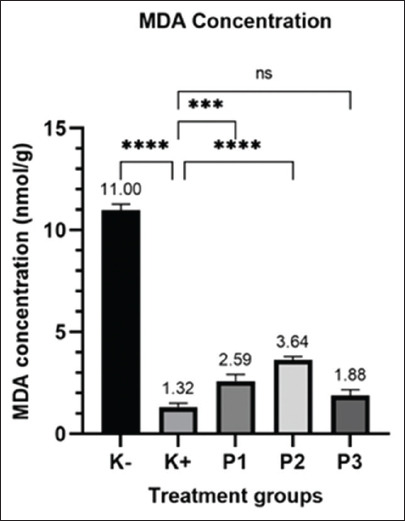
Measurement result of malondialdehyde concentration; ****: Significant difference, ns: no significant difference.

MDA levels measured using the competitive-ELISA method are presented in Figure-3. All three hydrolacin-gel treatment groups had MDA values of formula 1 (2.59 nmol/g), formula 2 (64 nmol/g), and formula 3 (1.88 nmol/g), which were close to those of the clindamycin control group with an MDA value of 1.31 nmol/g that was significantly different from that of the diabetic group (11 nmol/g). These results indicate that hydrolacin-gel has a potential effect to reduce oxidative stress due to inflammation induction in rats, with formula 3 being the most effective.

### Histopathological examination

**Figure-4:**
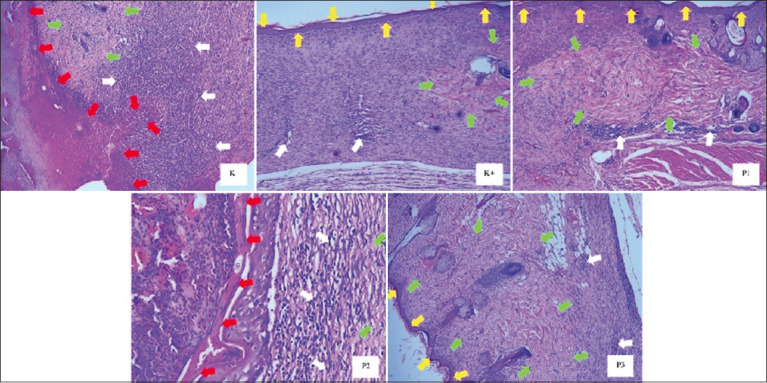
Results of histopathological examination (HE, 100×); red arrow: clotting area; yellow arrow: epithelium; white arrow: lymphocyte infiltration; green arrow: collagen tissue proliferation.

Microscopic observation of the wound healing process considers histopathological changes including cellular infiltration, collagen connective tissue production, neovascularization, and epithelial thickness. Figure-4 depicts the diabetic group (K-) with a wide clotting area (necrotic tissue area) on the skin surface accompanied by severe inflammation dominated by lymphocyte inflammatory cells and limited collagen connective tissue proliferation in the dermis layer. In contrast to the control group (clindamycin), the hydrolacin-gel formula 1 (P1) and formula 3 (P3) groups showed complete epithelial closure in the epidermal layer and collagen connective tissue proliferation in the dermis layer with good angiogenesis. However, small lymphocyte infiltration was still present in the dermis layer. However, group P2 (hydrolacin-gel formula 2) still showed several necrotic tissue areas.

**Figure-5:**
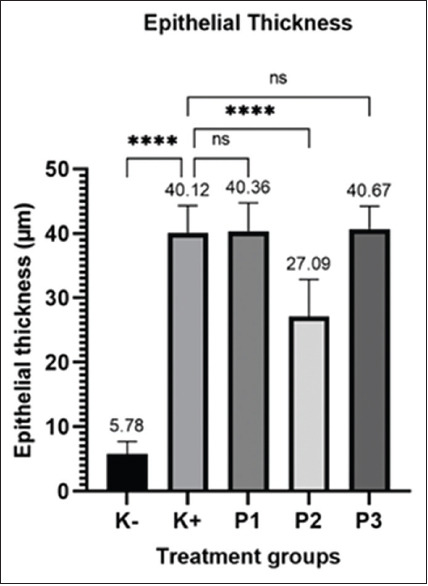
Measurement result of epithelial thickness; ****: significant difference, ns: no significant difference.

Significant differences in epithelial thickness were observed among the treatment groups (p < 0.05), as depicted in Figure-5. The average epithelial thickness in the diabetic (K group) group was 5.78 μm, which was significantly different from that in the K+ (clindamycin) group (40.12 μm, P1 [hydrolacin-gel formula 1], 40.36 μm, and 40.67 μm, respectively) and P2 (hydrolacin-gel formula 2) groups (27.09 μm, respectively). HE indicated that hydrolacin-gel triggers re-epithelialization and suppresses inflammation during wound healing in diabetic-infected MRSA.

## Discussion

The average droplet size of the curcuminoid nanoemulsion obtained in this study was 14.68 nm with a PdI value of 0.389, indicating that the curcuminoid nanoemulsion formula contains homogenously distributed nanosized particles. The PdI value of the nanoemulsion indicates the heterogeneity of a sample based on particle size, whereas the Z-average size distribution (droplet size) represents the average particle diameter. Nanoemulsion particle sizes typically range from submicron to approximately 5–200 nm, with a PdI value between 0.224 and 0.597 [17].

Curcuminoids have a potential effect against MRSA-infected wounds by inhibiting COX-2 and LOX in the inflammatory process [6]. Collagen contained in marine peptides can enhance angiogenesis and tissue repair [7]. Hydrolacin-gel was developed by combining curcuminoid compounds and CMPs. Curcuminoids act effectively against MRSA by targeting filamenting temperature-sensitive mutant Z, a pivotal molecule in bacterial cell division. They damage the bacterial cell wall, inhibit sortase A involved in bacterial adhesion to host cells, hinder bacterial adenosine triphosphate (ATP), enhance macrophage phagocytosis, and provide protection against pathogenic bacteria [18]. CMP collagen plays a crucial role in the three phases of wound healing. During the inflammatory phase, collagen aids in hemostasis and attracts macrophages through chemotaxis. During the proliferative phase, collagen forms folds for fibroblast integration, attracting fibroblasts to the wound area and acting as a model for new tissue growth. In the maturation phase, collagen provides strength to new tissue and enhances collagen fiber organization [19].

MDA, a common oxidation product of lipids, is often utilized as a biomarker of oxidative damage in the body [16, 20]. Increasing MDA levels in the body can be caused by chronic inflammation processes that can trigger prolonged lipid oxidation [16]. In our research, hydrolacin-gel containing curcuminoid and MDPs revealed the potential to reduce oxidative stress due to MRSA infection inflammation in diabetic rats. Hydrolacin-gel prevented the prolongation of the inflammatory phase, which is a part of the pathogenesis of chronic wound inflammation.

Wound recovery involves surface wound closure, repair of damaged blood vessels, regeneration of peripheral cells, and replacement of damaged muscle tissue with collagen fibers. Histopathological changes, including cellular infiltration, collagen tissue production, neovascularization, and epithelial thickness [21–23], are considered during microscopic observation of the wound healing process. The decrease in the number of inflammatory cell infiltration of wound skin tissues after treatment with hydrolacin-gel in this study indicates faster wound healing. Decreased inflammatory cell infiltration during wound healing indicates decreased inflammation. A faster reduction of inflammatory products suggests a quicker healing process [21, 23].

Wound healing involves repairing damaged blood vessels, regenerating peripheral cells, and replacing damaged muscle tissue with collagen fibers [22, 23]. The rate of re-epithelialization in skin tissue greatly influences the wound healing process. Re-epithelialization occurs through the mobilization, migration, mitosis, and differentiation of epithelial cells. Epithelial thickness reflects re-epithelialization speed. The faster the re-epithelialization process occurs, the faster the wound closure, thus accelerating wound healing [23]. The hydrolacin-gel group showed high collagen production, indicating a faster wound-healing process in the former group. The production of collagen during wound healing is crucial because it helps repair damaged or lost tissues. Collagen is a primary protein component of the extracellular matrix, and higher collagen formation indicates faster wound healing [21].

## Conclusion

Hydrolacin-gel combined with curcuminoid nanoemulsions and CMPs effectively inhibited the inflammatory process and increased re-epithelialization in MRSA-infected diabetic wound healing. Hydrolacin-gel with curcuminoid (130.2 mg) and CMPs (1.87 mg) at a concentration ratio of 2:1 appeared to be the best formula against MRSA infection in diabetic wounds.

## Authors’ Contributions

DASM, FDS, SAP, ABH, and SFT: Performed the experiment, data collection, data analysis, and drafted the manuscript. SIOS: Conceptualization, data analysis, supervision, and drafted the manuscript. All authors have read, reviewed, and approved the final manuscript.
